# Multimodality imaging in the detection and management of coronary and peripheral arterial disease in patients with cancer receiving cardiotoxic antineoplastic treatments: A clinical consensus statement of the ESC Council of Cardio-Oncology and the European Association of Cardiovascular Imaging (EACVI) of the ESC

**DOI:** 10.1093/ehjci/jeag103

**Published:** 2026-04-23

**Authors:** Giuseppina Novo, Teresa Lopez-Fernandez, Victoria Delgado, Patrizio Lancellotti, Ana G Almeida, Jutta Bergler-Klein, Jelena Celutkiene, Marc R Dweck, Marco Guglielmo, Geeta Gulati, Saeed Mirsadraee, Muhammad Sohaib Nazir, Alexander R Lyon, Ivan Stankovic

**Affiliations:** Department of Health Promotion, Maternal and Child Care, Internal Medicine and Medical Specialties (PROMISE), University of Palermo, Palermo, Italy; Division of Cardiology, University Hospital P. Giaccone, Palermo, Italy; Cardiology Department, Cardio-Oncology Unit, La Paz University Hospital, Idi PAZ Research Institute, Madrid, Spain; Heart Institute Hospital University Germans Trias i Pujol, Badalona, Spain; Centre of Comparative Medicine and Bioimaging, Institute of Research Germans Trias i Pujol, Badalona, Spain; Cardiovascular & Metabolism, Department of Cardiology, University of Liège Hospital, GIGA Institutes, CHU Sart Tilman, Liège, Belgium; Faculty of Medicine of Lisbon University, Cardiology, Heart and Vessels Department, CCUL@RISE, ULS Santa Maria, Lisbon, Portugal; Division of Cardiology, Department of Internal Medicine II, Medical University of Vienna, Vienna, Austria; Department of Cardiac and Vascular Diseases, Faculty of Medicine, Centre of Innovative Medicine, Vilnius University, Vilnius, Lithuania; BHF Centre for Cardiovascular Sciences, University of Edinburgh, Little France Crescent, Little France, Edinburgh, UK; Division of Heart and Lungs, Department of Cardiology, University Medical Center Utrecht, Utrecht, The Netherlands; Department of Radiology, University Medical Center Utrecht, Utrecht, The Netherlands; Division of Medicine, Department of Cardiology, Oslo University Hospital, Ullevål, Oslo, Norway; K.G. Jebsen Centre for Cardiac Biomarkers, Institute of Clinical Medicine, University of Oslo, Oslo, Norway; Department of Radiology, Royal Brompton Hospital, London, UK; National Heart and Lung Institute, Imperial College London, London, UK; School of Biomedical Engineering and Imaging Sciences, King's College London, London, UK; Cardio-Oncology Centre of Excellence, Royal Brompton Hospital, London, UK; Cardio-Oncology Centre of Excellence, Royal Brompton Hospital, London, UK; Department of Cardiology, Faculty of Medicine, Clinical Hospital Centre Zemin, University of Belgrade, Belgrade, Serbia

**Keywords:** coronary artery disease, peripheral artery disease, cardiotoxicity, cardio-oncology, echocardiography, computed tomography, cardiac magnetic resonance

## Abstract

Early detection of cancer and advances in treatment have significantly improved the survival rate of patients with cancer. Both cancer and its treatment can accelerate the onset of cardiovascular disease, adversely affecting prognosis of patients with cancer and survivors. Coronary artery disease (CAD) and peripheral artery disease (PAD) are common complications in patients with cancer. Cardiovascular imaging plays a central role in baseline risk assessment, detection, and treatment planning. The indications for the use of various imaging modalities are similar as in the general population. However, due to unique pathophysiological characteristics and clinical presentations of this population, the use of cardiac imaging in these vulnerable patients often needs to be adapted to the clinical circumstances and individual patient characteristics. In this clinical consensus statement, the European Society of Cardiology (ESC) Council of Cardio-Oncology and the European Association of Cardiovascular Imaging of the ESC have reviewed and summarized the current evidence in this field to aid clinicians in the selection of appropriate imaging modalities for the diagnosis, monitoring, and treatment of CAD and PAD in patients with cancer.

## Introduction

Significant improvements in cancer survival rates can be attributed to early detection and advances in treatment.^1^ Both cancer and its treatment can accelerate the onset of cardiovascular (CV) disease, adversely affecting the overall prognosis of cancer patients and survivors.^1,2^ Coronary artery disease (CAD) and peripheral artery disease (PAD) are common comorbidities in patients with cancer. Approximately 3% of patients with acute myocardial infarction (AMI) have cancer at the time of presentation, and a further 9% have a history of cancer.^3^ The association between cancer and vascular complications arises from shared risk factors (smoking, obesity, diabetes), the increased prothrombotic state frequently observed in cancer patients, as well as the action of antineoplastic drugs to cause or promote vascular damage through various mechanisms, including accelerated atherosclerosis, endothelial dysfunction, acute thrombosis, and microvascular injury.^3^

**Figure 1:**
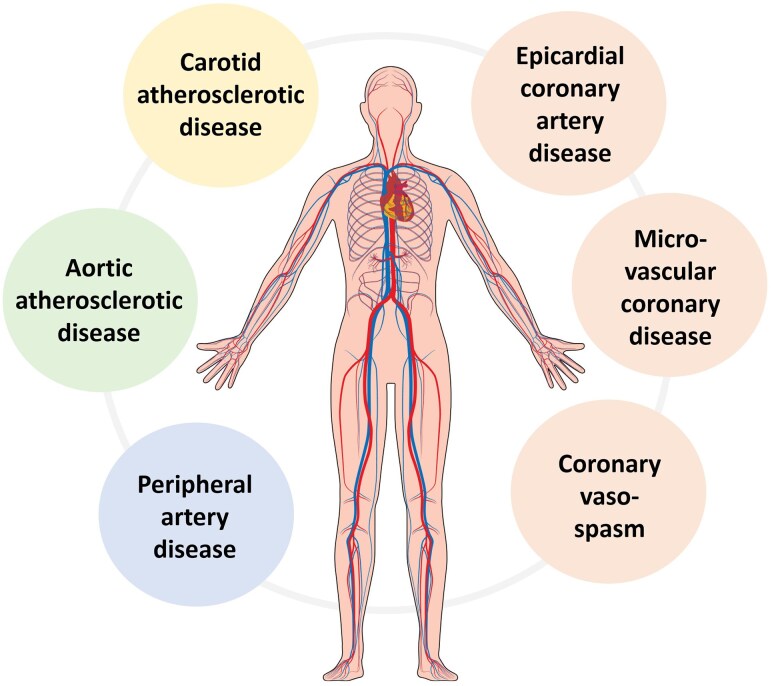
Antineoplastic treatments related vascular toxicity. Clipart credit: HAMED66261789, CC BY-SA 4.0.

Anticancer treatments, including cytotoxic chemotherapy, targeted therapies, immunotherapy, and radiotherapy (RT), can induce various clinical manifestations of vascular dysfunction or injury ranging from acute coronary syndromes (ACS; including AMI caused by coronary thrombosis, myocardial infarction with non-obstructed coronary arteries [MINOCA] or acute myocardial injury), chronic coronary syndromes (CCSs; including angina or ischaemia caused by coronary atherosclerosis or even structural or functional abnormalities in the absence of epicardial obstructive disease, angina with non-obstructive coronary arteries [ANOCA], ischaemia with non-obstructive coronary arteries [INOCA]), and peripheral vascular disease (PVD) (*Figure 1*).

While imaging is essential for the early detection and diagnosis of these vascular complications, aiding in differential diagnosis and guiding treatment strategies, the European Association of Cardiovascular Imaging (EACVI) survey on cardiac imaging in patients with cancer revealed significant heterogeneity in practices across various aspects of cardio-oncology.^4^ Due to the unique pathophysiology and clinical characteristics of patients with cancer, marked by increased frailty, complex comorbid profiles, and variable life expectancies, the diagnostic and therapeutic approaches for CAD and PAD in this population require careful adaptation from conventional clinical practices.^5^

The diagnosis is particularly challenging since cancer *per se* can be the source of several non-cardiac causes of acute and chronic chest pain, including pulmonary embolism, bone and chest wall metastases, pneumonia, and pleuritis. Additionally, cancer itself or cancer-related therapies may mimic or mask typical CAD symptoms; for example, epigastric pain resulting from mucositis may be mistaken for anginal pain, while cancer pain therapies may obscure anginal symptoms. Finally, chronically elevated troponin and/or creatine kinase levels, which sometimes occur in patients with cancer receiving treatment, may complicate the diagnosis of myocardial infarction. These patients are, therefore, often more complex to manage, resulting in prolonged stays in the emergency department or hospital.^3^

On the other hand, it is crucial to ensure that additional imaging tests, particularly in the context of CCS, do not delay the initiation of anticancer therapies. Balancing timely imaging with prompt therapy initiation is key to optimizing patient outcomes.

This clinical consensus statement, developed by the European Society of Cardiology (ESC) Council of Cardio-Oncology and the EACVI of the ESC, summarizes current evidence on the role of imaging in the management of cancer therapy–associated coronary and peripheral vascular complications. It aims to provide clinicians with advice on selecting optimal imaging modalities for the diagnosis, monitoring and treatment of CAD and PAD in patients with cancer. This clinical consensus statement, however, does not update or replace any ESC guidelines, but rather applies them to the specific context of cardio-oncology.

## Pathophysiology of vascular damage in patients with cancer receiving cardiotoxic antineoplastic treatments

**Figure 2:**
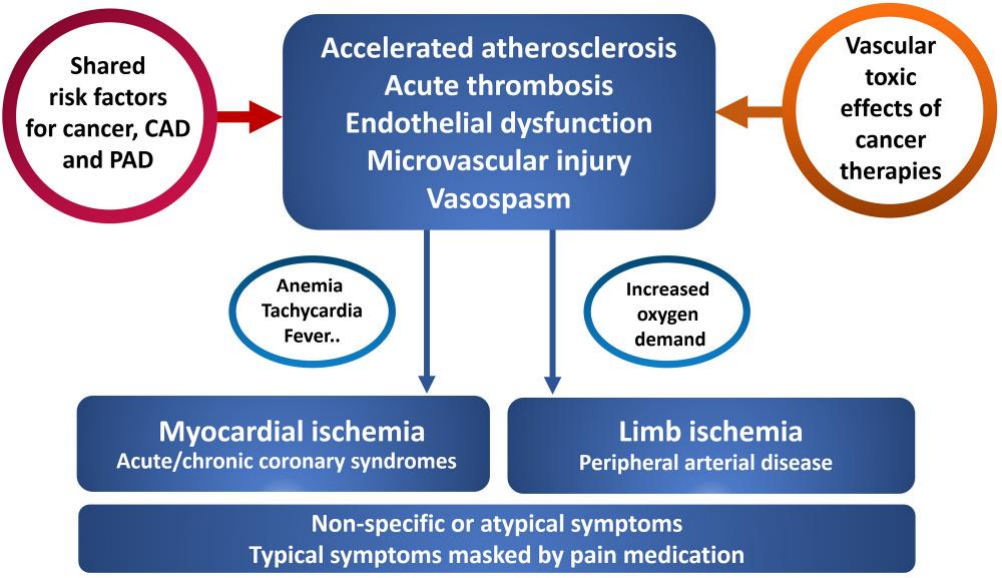
Interactions and overlaps between cancer, coronary artery disease (CAD), and peripheral artery disease (PAD). Patients with cancer are at an increased risk of developing CAD and PAD due to shared risk factors and the vascular toxic effects of cancer treatments. Cancer therapies can accelerate atherosclerosis, cause endothelial dysfunction, promote microvascular injury, and trigger acute thrombosis, and vasospasm. Both acute and chronic clinical manifestations of CAD and PAD in patients with cancer may be provoked or aggravated under conditions of increased oxygen demand (e.g. anaemia, tachycardia, fever). Diagnosis can be challenging, as patients with cancer may present with atypical symptoms, or their symptoms may be obscured by pain management or other treatments.

There are significant pathophysiological overlaps and interactions among cancer, CAD, and PAD beyond the increased risk resulting from the common shared risk factors such as age, smoking, obesity, diabetes mellitus, dyslipidaemia, hypertension, and a sedentary lifestyle (*Figure 2*). Cancer therapies may promote atherosclerosis, induce vasospasm, and acutely or chronically worsen CAD and PAD through different mechanisms.^6,7^ A common factor is cancer-related anaemia which may increase susceptibility to type 2 myocardial infarction (MI) or aggravate limb ischaemia. Many antineoplastic drugs are associated with an increased risk of vascular damage.^8–34^  *Table 1* summarizes the main antineoplastic drugs related to the occurrence of acute and CCSs, peripheral arterial disease, and Raynaud’s phenomenon. Raynaud’s phenomenon is also a common toxicity after antineoplastic treatment with bleomycin, cyclophosphamide, platinum compounds, vinca alkaloids, and fluoropyrimidines, especially if used in combination.^31^ Damage can occur acutely and at later stages of treatment^8–34^

**Table 1:** Pathophysiological mechanisms leading to coronary and peripheral artery toxicity related to antineoplastic drugs

Drug	Acclerated atherosclerosis	Vasospasm	Microcirculatory dysfunction	Acute thrombosis	Temporal occurrence^a^	Specific considerations
Antimetabolites		X			5-FU: 2–5 days into first course^9^	Fluoropyrimidines can be administered as a bolus, slow infusion (5-FU) or orally (capecitabine) Direct endothelial cell damage from toxic metabolites and coronary vasospasm are thought to play roles in FU induced cardiotoxicity^16^ A pooled analysis of over 25 000 patients found FU-associated ischaemic event rate of 2.24% and mortality of 0.12%^10^ The most frequent causes of cardiotoxicity-related death were sudden cardiac arrest (28.95%) and myocardial infarction (27.19%)^10^
Alkylating agents		X	X	X	Cyclophosphamide: 1–10 days after the first dose Cisplatin: within hours of completion of infusion^9^	Cisplatin-induced cytotoxicity is related to increased generation of endothelial micro- particles, impaired NO-dependent vasodilation and oxidative stress leading to prothrombotic effects, microvascular dysfunction and vasospasm^6,25,26^
Antimicrotubule Agents		X	X		Paclitaxel: Within 1 h into infusion to 14 days Vinca alkaloids: hours to 3 days^9^	
VEGFi	X	X	X	X	Initial high risk window during the first 6 months, long term late effects may also occur^15^	
Nilotinib, ponatinib	X		X	X	Within the first 6 months of treatment, but event after long term exposure (5 years)^12^	Second and third generation TKIs, particularly nilotinib and ponatinib, can cause adverse CV events, such as MI, stroke and especially PAD These events arise from the inhibition of kinases not involved in the pathogenesis of the tumour disease, so called off-target effects^14,26^
Erlotinib				X	After 3-month erlotinib treatment^5^	
ICI	X	X		X	Initial high risk window during the first 3 months of treatment, long term late effects, after 6 months, may also occur^5^	Fulminant myocarditis is the most feared form of ICI related CV toxicity^17^ ICI-induced myocarditis can occur as part of the myocarditis-myositis-myasthenia gravis overlap syndrome Incidence of ACS during ICI therapy ranges from 0.003% to 1%^18,19^ ICI can also accelerate peripheral atherosclerosis and increase the rate of MACE. Proposed mechanisms of ICI mediated damage include plaque destabilization, inflammatory coronary artery vasculitis and vasospasm^20–26^ Acral vascular syndromes requiring finger amputation have been rarely reported during treatment with ICIs, such as nivolumab^24^
IMiD				X	Most cardiac events occur during the first 1–3 months of treatment^13^	
PI		X		X	Within the first 3 months of therapy^16^	
ADT	X				Initial high risk window during the first 6 months of therapy^15^	GnRH agonists are linked to an increased risk of ACS and CV mortality GnRH antagonists are less cardiotoxic than GnRH agonists^34,35^ The second generation androgen deprivation therapy Enzalutamide and the androgen metabolism inhibitor Abiraterone may also increase the risk of CAD^5^ Proposed mechanisms include elevation of LDL cholesterol, development of insulin resistance and type 2 diabetes mellitus, and T-cell activation in plaques triggering rupture
Radiotherapy	X		X		From 5 years after exposure to even 20 years^12^	

ACS, acute coronary syndrome; ADT, androgen deprivation therapy; CAD, coronary artery disease; CV, cardiovascular; FU, fluorouracil; GnRH, gonadotropin-releasing hormone; ICI, immune checkpoint inhibitors; IMiD, immunomodulators; LDL, low-density lipoprotein; MACE, major adverse cardiovascular events; PAD, peripheral artery disease; PI, proteasome inhibitors; TKI, tyrosine kinase inhibitors; VEGFi, vascular endothelial growth factor inhibitors.

^a^of cardiotoxic effect after administration.

Radiation therapy poses significant long-term risk of complications, particularly to the vascular structures within the irradiated field, including the coronary arteries, carotid arteries, aorta, renal arteries, and heart valves.^32^ This risk is dose-dependent and symptoms generally appear years after treatment.^33,34^ Radiation induced damage is primarily mediated by endothelial injury, inflammation, vascular wall fibrosis, and subsequent arterial stenosis.^34^ Historically, RT used higher doses and less targeted techniques, leading to more widespread and significant cardiotoxicity and vascular toxicity. Whilst advancements in RT techniques have reduced radiation-induced CV toxicity but it remains an ongoing concern, particularly in cancer patients with pre-existing atheroma which can be accelerated by radiation therapy and associated oxidative stress. These high CV risk cancer patients requires optimal CV risk factor control and careful monitoring.^35^

The presence of CV risk factors and pre-existing CAD predispose patients to these complications, both during or after cancer treatment. Therefore, prior to initiating thoracic RT or other antineoplastic treatment associated with CAD events, it is crucial to carefully stratify CV risk in primary prevention using currently available risk scores (e.g. SCORE 2 and SCORE 2 old patients) and where available imaging evidence of coronary atherosclerosis to effectively manage CV risk factors and optimize the treatment of pre-existing CAD.^5^

Patients with cancer are at an increased risk of developing CAD and PAD due to shared risk factors and the vascular toxic effects of cancer treatments. Cancer therapies can accelerate atherosclerosis, cause endothelial dysfunction, promote microvascular injury, and trigger acute thrombosis and vasospasm. Both acute and chronic clinical manifestations of CAD and PAD in patients with cancer may be provoked or aggravated under conditions of increased oxygen demand (e.g. anaemia, tachycardia, fever). Diagnosis can be challenging, as patients with cancer may present with atypical symptoms, or their symptoms may be obscured by pain management or other treatments.

## Baseline imaging in patients with cancer

Similar to the general population, all non-invasive cardiac imaging modalities play a crucial role in detecting and monitoring CAD and PAD. However, specific considerations apply to patients with cancer undergoing cardiotoxic anticancer treatments (*Table 2*). The following sections discuss the use and role of cardiac imaging across various clinical scenarios in patients with cancer.

**Table 2:** Summary of potential differences in the use of cardiovascular imaging for coronary artery disease in patients with and without cancer

	Patients without cancer	Patients with cancer
General principles		
Pre-test probability	Pre-test probability based on age, sex, symptoms and risk factors	Pre-test probability may be underestimated due to accelerated atherosclerosis^6^
Pathophysiology	Classic mechanisms (plaque rupture/erosion) more frequent	Cancer therapy-related mechanisms (vasospasm, thrombosis, MVD, therapy-related injury) may co-exist with atherosclerosis^3^ MINOCA is more prevalent in patients with cancer
Constraints	Usually fewer limitations	Frailty, possible interference with the timing of cancer therapy (imaging tests with long wait times)
Imaging modality-specific considerations		
Echocardiography	First-line imaging tool Stress echocardiography with standard advantages and limitations	Bedside assessment of critically ill cancer patients Poor acoustic windows, even with use of contrast agents in some patients (e.g. prosthesis implantation in breast cancer patients) Exercise echocardiography may not be feasible due to low performance status in advanced-stage cancer
Cardiac CT (CCTA and CAC)	Rule out obstructive CAD in low–intermediate risk patients CAC scoring for risk stratification and prevention	Simultaneous assessment of other important pathologies (e.g. pericardial involvement, masses) CCTA facilitates decision-making in patients treated with fluoropyrimidines who develop chest pain by excluding or confirming obstructive coronary lesions CCTA may help avoid invasive coronary angiography in frail patients CAC can be assessed on routine scans performed for tumour staging
CMR	Tissue characterization (scar, viability)	Differentiation between ICI-related myocarditis and myocardial ischaemia Simultaneous assessment of other important pathologies related to cancer (e.g. pericardial involvement, masses)
Nuclear imaging	SPECT is widely used for ischaemia detection	SPECT accuracy may be reduced by prior chest radiation and attenuation artefacts

CAC, coronary artery calcification; CCTA, coronary computed tomography angiography; CMR, Cardiac magnetic resonance; ICI, immune checkpoint inhibitor; MINOCA, myocardial infarction with non-obstructive coronary arteries; MVD, microvascular disease; SPECT, single-photon emission computed tomography

Baseline CV risk assessment before starting cardiotoxic cancer therapies should include Heart Failure Association—International Cardio-Oncology Society (HFA-ICOS) risk stratification (Class IIa, Level C) and a 12-lead electrocardiogram (ECG) for all patients starting potentially cardiotoxic cancer therapy, as recommended in the 2022 ESC cardio-oncology guidelines.^5^ According to the same ESC guidelines, baseline transthoracic echocardiography (TTE) is recommended in patients at high or very high risk of CV toxicity (Class I, Level C).^5^ For treatments mainly associated with vascular toxicity and not captured by the HFA-ICOS score (e.g. androgen deprivation therapy [ADT], immune checkpoint inhibitors [ICIs], alkylating agents, and fluoropyrimidines), baseline TTE should be driven by patient CV risk level or the presence of pre-existing CV disease rather than performed routinely. The 2022 ESC cardio-oncology guidelines do not define an indication for routine baseline stress echocardiography, and there is no evidence supporting its systematic use beyond standard indications in the general population^5^ (see Section Functional Imaging).

If echocardiography is non-diagnostic, then cardiovascular magnetic resonance (CMR) should be considered (Class II, Level C) for the assessment of cardiac function.^5^

Incidental coronary artery calcification (CAC) detected on routine thoracic staging computed tomography (CT), when available, can improve CV risk stratification and might be useful especially in patients with cancer treated with antineoplastic treatments burdened by direct vascular toxicity (e.g. vascular endothelial growth factor inhibitors [VEGFi], ADT, some second- and third-generation BCR-Abl tyrosine kinase inhibitors [TKI], and RT to a field including the heart and coronary arteries) and a good prognosis (>6 months).^36–38^

This is particularly relevant in cancer patients with low and intermediate risk scores, which have underestimated their atherosclerotic burden.^37,39^ Although oncology CT scans are usually not ECG-gated, CAC presence/absence can still be assessed, providing an estimate of coronary atherosclerotic plaque burden that may help reclassify CV risk. According to the 2024 ESC guidelines for the management of CCSs,^39^ when CAC findings are available from previous chest CT scans, using these findings to enhance risk stratification and guide treatment of modifiable risk factors should be considered (Class IIa, Level of Evidence C).

**Figure 3:**
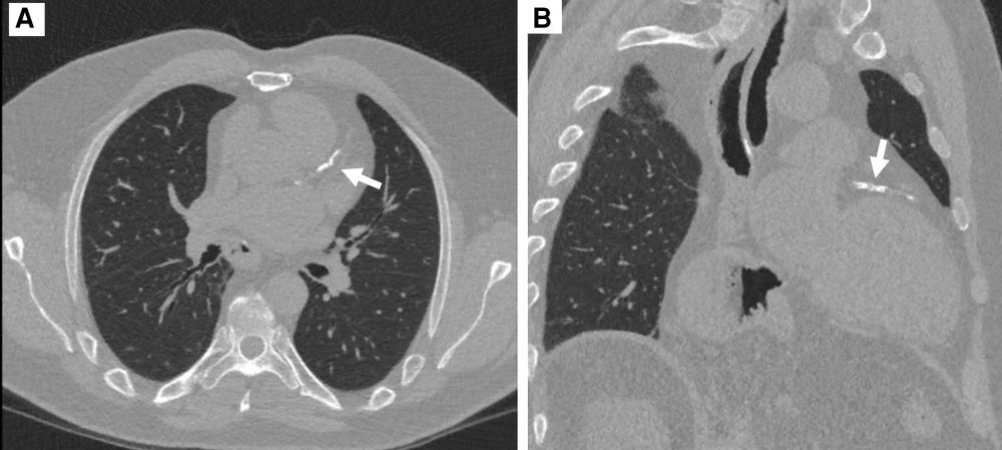
Thoracic computed tomography (CT) in a 52-year-old male with colon adenocarcinoma. The CT scan performed for tumour staging revealed extensive calcification of the left anterior descending coronary artery.

For example, the absence of coronary calcium has a high negative predictive value for obstructive CAD and may support a conservative approach with clinical follow-up, whereas any coronary artery calcium score (CACS) >0 can shift patients across risk thresholds into a higher-risk category and thereby support decisions on initiating preventive therapy.^39–42^  *Figure 3* shows an example in which calcium score evaluation in a routine staging CT scan was helpful to reclassify the patient’s CV risk.

In patients with cancer with CACS >0 on routine CT performed for staging cancer, no specific evidence is available regarding the indication to further assess ischaemia; however, according to this task force, the performance of tests for ischaemia detection, beyond primary prevention strategies, is not needed in the absence of significant cardiac symptoms. In selected cancer patients with a good prognosis, a dedicated CT coronary angiogram can be helpful to define the extent and location of the CAD.

It is essential that the waiting time for cardiac imaging does not lead to a delay or postponement of cancer treatments, as this would have potentially impacted the overall prognosis of the patient. Therefore, it is crucial to carefully consider the incremental value and clinical implications of any CV imaging test and how rapidly it can be performed and reported.

Assessment for PAD was not listed in the baseline CV assessment requirement in the 2022 ESC cardio-oncology guidelines.^5^ Recognition of pre-existing vascular disease (including PAD) falls under overall CV risk stratification as part of the baseline risk profile. However, targeted screening for pre-existing PAD (i.e. ankle-brachial index measurement) in addition to clinical examination, may be considered in subgroups of cancer patients receiving specific agents that have been associated with a high vascular toxicity (second-generation tyrosine kinase inhibitors like nilotinib or ponatinib) to identify patients who may benefit from more strict monitoring (every 6 months) during and after cancer treatment.^5^

## Acute coronary syndromes

ACS are a common and increasingly encountered form of cancer therapy–related CV toxicity (CTR-CVT), significantly affecting the overall outcome of patients with cancer.^3^ Several mechanisms can lead to ACS in patients with cancer, including the cardiotoxic effects of chemotherapy, targeted therapy and RT.^43,44^

Cancer itself may predispose to coronary plaque erosion and acute thrombosis (Type 1 MI). Optical coherence tomography studies in patients with a cancer history and ACS have revealed a higher incidence of thin cap fibroatheroma and high-risk plaque features.^44^

Although patient characteristics often differ between those managed invasively and those treated conservatively, introducing potential selection bias, the 2022 ESC Cardio-Oncology guidelines emphasize prompt invasive coronary angiography in patients with cancer and ACS who have a reasonable expected prognosis (>6 months).^5^

The diagnosis of ACS in patients with cancer follows the same criteria as in patients without cancer.^45^ It is important to recognize that symptoms in patients with cancer may be more atypical or non-specific. Moreover, symptoms such as chest pain, nausea, fatigue, or dyspnoea can often be attributed to the cancer or its treatment. Consequently, a low threshold for performing an ECG and cardiac troponin is advised, along with non-invasive imaging to confirm the diagnosis.^46^

### Echocardiography

Echocardiography plays an essential role in the early risk stratification of patients with suspected ACS, especially when clinical symptoms and ECG findings are inconclusive.^46^ In patients with ST-elevation ACS, TTE can detect regional wall motion abnormalities, identify mechanical complications, and help exclude alternative diagnoses. In patients with non-ST elevation myocardial infarction, echocardiography, in the early stages following presentation, can provide rapid assessment of patients with suspected Takotsubo syndrome, acute heart failure, major pulmonary embolism, severe valvular heart disease, and cardiac tamponade.

### Coronary computed tomography angiography

Coronary computed tomography angiography (CCTA) provides a non-invasive, high-resolution anatomic evaluation and direct visualization of CAD.^47^ Unlike invasive coronary angiography, CCTA provides rapid, non-invasive visualization of both obstructive and non-obstructive CAD, allowing for high-resolution anatomic evaluation.^48^ According to the 2023 ESC Guidelines on the management of acute coronary syndromes,^45^ in patients with suspected ACS, normal (or uncertain) high sensitivity troponin levels, no ECG changes, and no recurrence of pain, incorporating CCTA as part of the initial work-up should be considered. CCTA can inform the management of patients with cancer and suspected ACS across various clinical scenarios. For instance, by ruling out coronary plaques in patients with suspected fluoropyrimidine-induced coronary vasospasm, CCTA enables the continuation of cancer therapies by using long-acting nitrates and vasodilating calcium channel blockers, or alternative agents such as S1-based therapy instead of fluoropyrimidines,^5,49^ Additionally, CCTA can detect non-obstructive atherosclerotic plaques that may benefit from preventive medical therapies as well as high-risk coronary stenosis, thrombus, and plaque formations with further management tailored to the patients’ prognosis and life expectancy.^5^

### Cardiac magnetic resonance imaging

**Figure 4:**
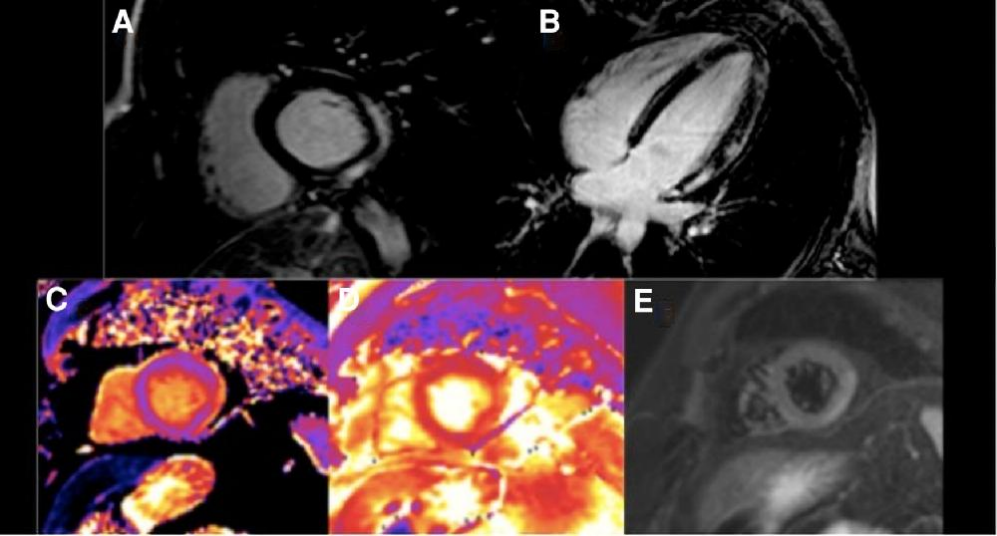
Cardiovascular magnetic resonance images in a 44-year-old woman with a metastatic breast cancer and immune checkpoint inhibitor-associated acute myocarditis, non-ischaemic subepicardial late gadolinium enhancement in the inferior and inferolateral walls: (*A*, *B*); concomitant myocardial oedema demonstrated by elevated native T1 (1280 ms) on T1 mapping (*C*); elevated T2 (72 ms) on T2 mapping (*D*); high signal intensity on T2 STIR (E).

CMR is particularly useful in patients presenting with a working diagnosis of MINOCA.^45^ Here it can help differentiate true myocardial infarction from other differential diagnoses including Takotsubo syndrome and myocarditis, by elucidating regional myocardial abnormalities, and by detecting different patterns of oedema and myocardial damage with late gadolinium enhancement (LGE). In case of myocardial infarction, LGE usually affects the subendocardium, which is typically spared in myocarditis.^48^ In patients with Takotsubo syndrome, LGE is often absent or patchy and does not correspond to the extent of the wall motion abnormalities.^50^  *Figure 4* represents the case of a patient in which CMR was decisive to diagnose ICI myocarditis.

### Nuclear techniques

Nuclear perfusion imaging may be helpful in selected stable patients presenting with atypical chest pain or dyspnoea and normal troponins and ECG. Here it can serve as an alternative to CCTA or CMR stress testing when these are contraindicated or not available when additional functional assessment is needed.^51^ Myocardial perfusion imaging (MPI), using single photon emission computed tomography (SPECT) or positron emission tomography (PET), can rapidly reveal fixed perfusion defects and reversible myocardial ischaemia.^52^ Furthermore, 18F-fluorodeoxyglucose PET may provide complementary diagnostic information to CMR in challenging cases of suspected ICI myocarditis,^53^ although due to possible pitfalls, this technique is advised only in settings with high expertise.

Key points

- For patients with cancer with suspected ACS, the same diagnostic pathway applies as for patients without cancer.
- Non-invasive cardiac imaging is helpful in patients with cancer and suspected ACS when symptoms, biomarkers/ECG or clinical findings are equivocal and do not clearly indicate the need for immediate invasive coronary angiography.
- Echocardiography is the first-line technique to detect new LV wall motion abnormalities, support the diagnosis, assess haemodynamics, and exclude alternative diagnoses.
- CCTA can be useful in stable patients with suspected non-ST elevation ACS at low clinical risk to exclude coronary atherosclerosis and to characterize obstructive and non-obstructive disease, helping guide subsequent management
- CMR is particularly useful for clarifying the underlying diagnosis, in haemodynamically stable patients, especially when myocarditis, Takotsubo syndrome, or MINOCA is suspected.
- Nuclear perfusion imaging may be useful in selected stable cases when additional functional assessment is needed (e.g. for differential diagnosis in CTR-CVT), but it is not helpful for the acute/unstable patient.

## Chronic coronary syndromes

Several cancer treatments may increase the risk of CCS through different pathophysiological mechanisms. In patients with cancer two main scenarios may arise: patients with pre-existing CAD who develop new symptoms during cancer treatment, and patients without known CCS who experience new symptoms. In clinical practice, chest pain or angina may be caused by: (a) obstructive epicardial CAD; (b) vasospasm or functional/structural microvascular alterations without epicardial obstructive CAD (ANOCA/INOCA); (c) post-ACS or after revascularization; and (d) heart failure of ischaemic or cardiometabolic origin.^39^

**Figure 5:**
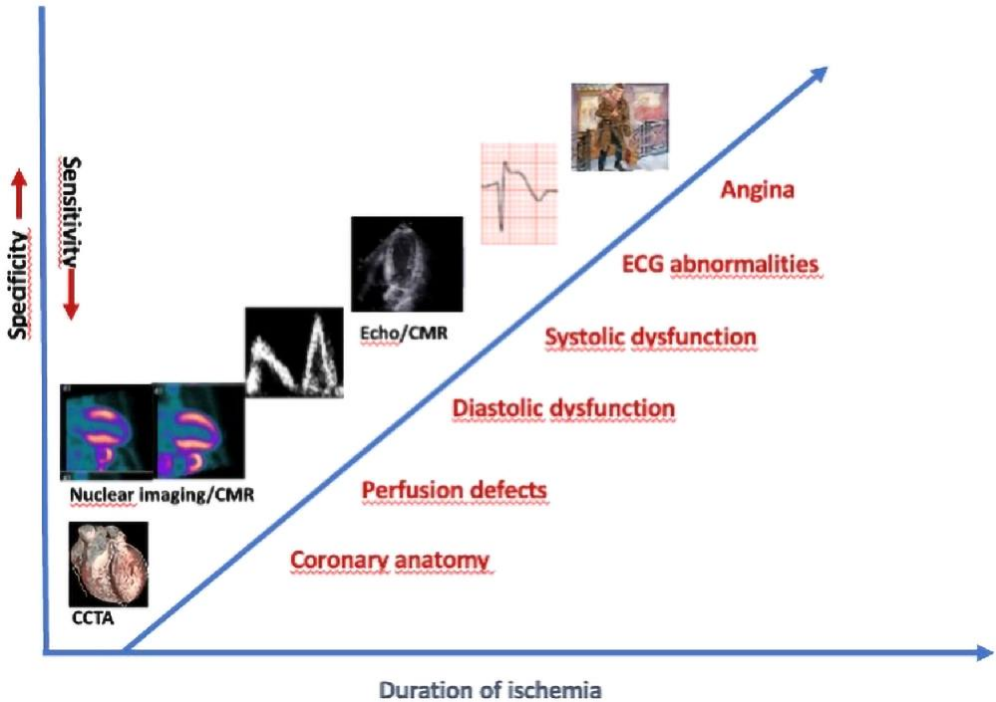
Sensitivity and specificity of the different imaging techniques in the context of ischaemic cascade.

When patients receiving cancer therapy present with chest pain or other concerning symptoms, a careful clinical evaluation should be performed. The diagnosis of CAD should adhere to the 2024 ESC Guidelines for the diagnosis and management of CCS, which outline the evaluation of clinical likelihood of obstructive CAD.^39^ For patients with limited life expectancy, severe frailty, and low quality of life, where revascularization may be deemed futile, CCS can be diagnosed clinically, and managed with medical therapy and lifestyle modifications. If there is uncertainty regarding the diagnosis of CCS in these patients, it is reasonable to establish a diagnosis using non-invasive functional imaging before initiating treatment. *Figure 5* shows the sensitivity and specificity of the different imaging techniques in the context of ischaemic cascade.

### Non-invasive cardiac imaging

Patients with a very low clinical likelihood of CAD (≤5%) generally do not require imaging evaluation. For those with a very high clinical likelihood of CAD (>85%), invasive coronary angiography is considered the appropriate first-line test for suspected CCS unless deemed futile due to clinical conditions.^39^ For patients with an intermediate diagnostic probability, non-invasive anatomical or functional diagnostic tests are typically advised. Ultimately, the decision for additional testing is guided by clinical assessment, available resources, local expertise, patient-specific factors, and individual preferences. Overall, these tests provide prognostic information by predicting the risk of future adverse events and can serve as complementary tools in cases of inconclusive findings or when assessing the need for revascularization.

Echocardiography is the first-line technique for assessing ventricular function and wall motion abnormalities. In the absence of obvious wall motion abnormalities, a decreased global longitudinal strain and particularly regional strain abnormalities can strengthen the suspicion of CAD. However, one should be aware that global longitudinal deformation can be impaired by other common confounding conditions such as hypertension and diabetes beyond cancer therapy. According to clinical suspicion, further functional or anatomical imaging tests are advised to confirm a diagnosis.

#### Anatomical imaging

CCTA is the preferred method for ruling out CAD in patients with a low (5–15%) to moderate (15–50%) clinical likelihood of CCS due to its high negative predictive value. Furthermore, CCTA can be seamlessly integrated with cardiac imaging sequences into routine CT scans performed for tumour staging, providing a dual assessment with minimal additional radiation exposure. CCTA provides the only method to detect both obstructive and non-obstructive coronary plaque and also identifies atherosclerotic plaque characteristics, such as calcified, non-calcified, and high-risk plaques. The detection of obstructive and non-obstructive CAD with CT allows for the prescription of better targeted preventive therapies and better outcomes compared to standard care.^54^ Additionally, CCTA can quantify the burden of coronary atherosclerotic plaque^55^ and evaluate the peri-coronary fat attenuation index, a surrogate marker of inflammation, that both appear to provide additional prognostic information.^56^ CT scans without the use of contrast agents enables not only the assessment of CAC, but also simultaneous evaluation of the pericardium, providing excellent visualization of pericardial calcification, which may be seen in patients with cancer following RT. Non-contrast CT attenuation measurements can also characterize pericardial fluid, aiding in the identification of malignant or haemorrhagic effusions.^57^

Photon-counting detector computed tomography (PCD-CT) offers significant advancements over conventional energy-integrating detector CT, including better spatial resolution, artefact reduction, and spectral analysis. In cardiac imaging, PCD-CT can offer a more accurate assessment of coronary artery stenosis and plaque characterization. Additionally, it might improve the visualization of myocardial fibrosis through late enhancement imaging assessment and extracellular volume measurements.

The use of PCD-CT in cardiac imaging holds significant potential, positioning itself as a valuable modality that could serve as a one-stop-shop by integrating both angiography and tissue characterization into a single examination.^58^ However, despite its potential, large-scale clinical trials, standardization of protocols and cost-effectiveness considerations are required for the broader integration of PCD-CT into clinical practice.

Furthermore, it does not provide information about the haemodynamic significance of the coronary stenosis requiring additional functional tests to identify ischaemia.

**Figure 6:**
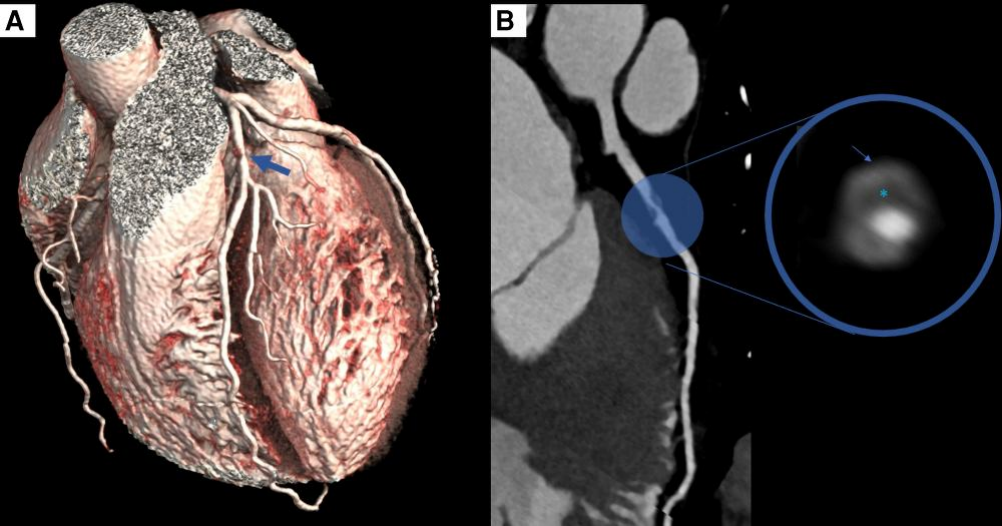
Cardiac coronary computed tomography angiography in a 47-year-old female patient with atypical chest pain and mammary tumour. Severe stenosis of the left anterior descending coronary artery is demonstrated on three-dimensional volume-rendered reconstruction (*A*, arrow) and multiplanar reconstruction (*B*). The stenosis is caused by a plaque exhibiting high risk features, with the ‘napkin-ring sign’, a low attenuation plaque (*B*, asterisk) with a hyperdense cap (*B*, arrow).

*Figure 6* shows an example of coronary stenosis detected and characterized by CCTA in patient with atypical chest pain and mammary tumour.

#### Functional imaging

**Figure 7:**
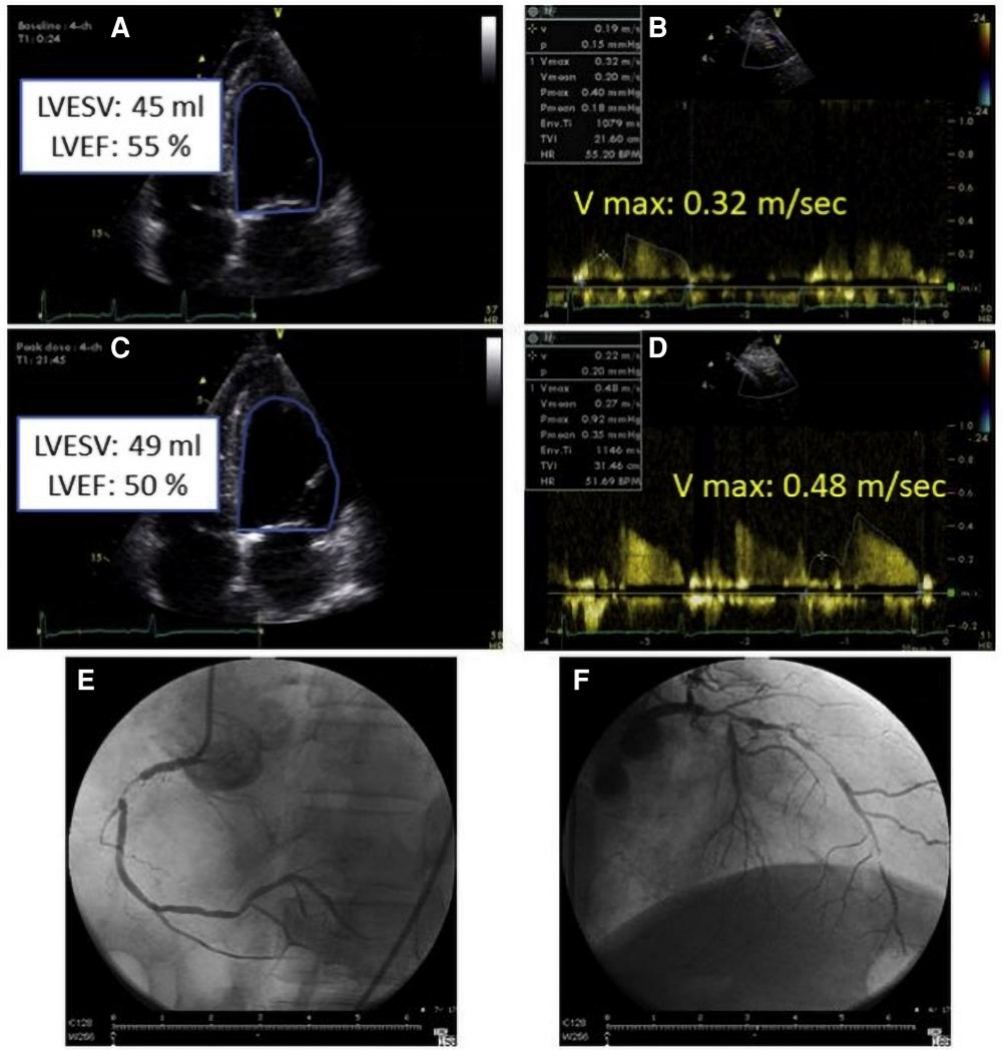
A 68-year-old woman with Hodgkin lymphoma localized in the mediastinum, treated with 12 cycles of doxorubicin, bleomycin, vinblastine, and dacarbazine and radiotherapy, referred with effort chest pain for dipyridamole stress echocardiography. (*A*) Resting four-chamber view showed preserved left ventricular (LV) ejection fraction and no wall motion abnormalities. (*B*) Resting coronary flow in the distal left anterior descending coronary artery (LAD) was 0.32 m/s. (*C*) Peak-stress four-chamber view showing LV systolic enlargement and diffuse wall motion abnormalities. (*D*) Peak coronary flow in the distal LAD was 0.48 m/s, and coronary flow reserve was 1.5. (*E*, *F*) Coronary angiography showed multivessel disease with critical obstruction of the three main vessels.

Functional imaging tests are advised for diagnosing and quantifying myocardial ischaemia or scar in patients with a moderate or high likelihood of CCS (15–50% or 50–85%, respectively), and those with known CAD. They may also be preferred in patients with a very high burden of CAC on routine non-gated staging CT scans. Stress echocardiography provides a highly specific assessment of myocardial ischaemia and allows the evaluation of myocardial viability and contractile reserve. Whilst sensitivity is lower, it also enables the evaluation of valve disease and the detection of heart failure with preserved ejection fraction.^59^ Stress echocardiography using exercise is often preferred because of its prognostic value, but it may not always be feasible in patients with cancer with limited performance status. In these cases, pharmacological stress agents such as adenosine, regadenoson, dobutamine, or dipyridamole can be used. Evaluation of coronary flow reserve (CFR) in the left anterior descending coronary artery can further improve the diagnostic accuracy of stress echocardiography in the general population; however, the reliability of this method is heavily influenced by training and expertise (*Figure 7*). Moreover, patients with breast cancer, who have undergone left breast prosthesis implantation, may exhibit poor acoustic windows, even with the use of contrast agent.

CMR provides a comprehensive evaluation of CAD by offering detailed insights into cardiac anatomy, function, and myocardial scar. Additionally, similar to CT, CMR provides detailed information regarding pericardial involvement that could be caused by antineoplastic treatment and particularly RT. It can assess pericardial thickening, identify active pericardial inflammation, and quantify and characterize pericardial effusions. Additionally, CMR has demonstrated excellent diagnostic accuracy for the non-invasive detection of constrictive physiology.^60^

**Figure 8:**
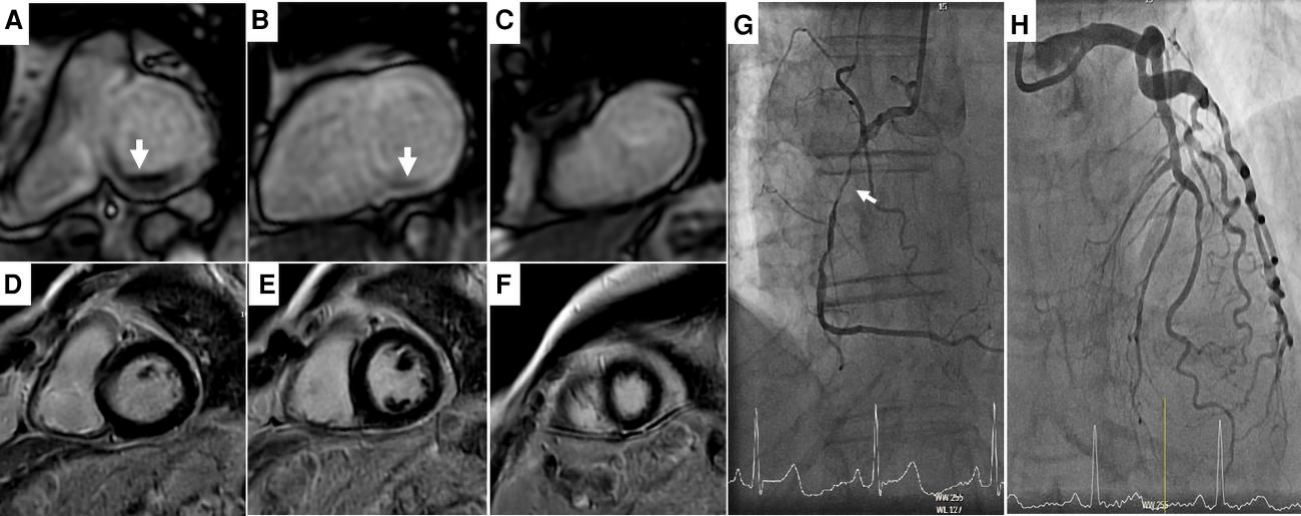
Stress cardiovascular magnetic resonance imaging in a 68-year-old male patient with atypical chest pain and a history of lung carcinoma treated with chemotherapy and radiotherapy. Perfusion images (*A*, *B*, *C*) acquired after the administration of adenosine demonstrate a large perfusion defect in the basal inferior wall and basal inferior interventricular septum (*A*), as well as a smaller defect in the middle portion of the inferior wall. Late gadolinium enhancement images (*D*, E, *F*) reveal no evidence of prior myocardial infarction. Invasive coronary angiography (*G*) showed subtotal occlusion of the mid-right coronary artery (arrow).

Stress CMR has emerged as a robust functional test, demonstrating high diagnostic accuracy in identifying obstructive and non-obstructive CAD in both known and suspected cases^61^ (*Figure 8*). In addition, stress CMR provides important prognostic information.^62,63^ An emerging application of stress CMR is the quantification of myocardial blood flow (MBF), which enhances the assessment of the burden of myocardial ischaemia.^64^ Additionally, the implementation of these advanced sequences can aid in the detection of cardiac microvascular disease.^64^ While the use of stress CMR may be influenced by factors such as availability, acquisition times, the presence of cardiac implantable electronic devices, and the need for patient cooperation, it is becoming increasingly accessible and feasible for most patients, except those who are critically ill, where alternatives like stress echocardiography may also present challenges. Higher sensitivity CMR techniques including fast SENC (Myostrain) can assess for CV causes of chest pain with high sensitivity. Importantly, the majority of magnetic resonance imaging (MRI)-compatible or MRI-conditional devices can now safely undergo CMR in centres with the necessary expertise. Furthermore, with experience, stress CMR can typically be completed in about 30 min, making it comparable in duration to a dobutamine stress echocardiography.

Stress SPECT is highly sensitive for detecting ischaemia and is readily available, but not as specific as stress echocardiography. Quantitative estimates of CFR can be derived from SPECT myocardial perfusion images. However, this method underestimates CFR, particularly at high flow rates.^65^

PET provides reference standard assessments of myocardial perfusion, with the major advantage of allowing flow quantification that helps identify patients with balanced ischaemia due to both advanced obstructive disease and the microvascular dysfunction that is frequently observed in patients with cancer, particularly in those post-RT.^35^ Limitations of PET-CT arise from its limited availability compared with other imaging modalities and relatively high costs and radiation.

Fractional flow reserve derived from CT (FFR-CT) can be estimated non-invasively by applying computational fluid dynamics to anatomical image data obtained from CCTA. This process does not require additional CT imaging, modifications to acquisition protocols, or the administration of extra medications.^66^ FFR-CT can help in the diagnosis of obstructive CAD, although it can only be performed on scans with excellent image quality, and analysis is often expensive to perform. Stress CT perfusion (CTP) is a MPI technique that evaluates the distribution of MBF during both rest and hyperaemia, providing valuable insights into the presence of myocardial ischaemia. Unlike FFR-CT, stress CTP requires the use of additional contrast agents and exposes patients to radiation beyond the baseline CCTA study.^67^ Furthermore, stress CTP necessitates the use of advanced-generation scanners and is currently of limited availability in clinical practice.

Key points

- In the setting of CCS, two main scenarios emerge: patients with known CAD who develop new or worsening symptoms during cancer treatment, and patients without prior CAD who present with new symptoms, potentially treatment-related.
- In patients with active cancer and no previous CCS, symptoms can be atypical; a high clinical suspicion is warranted, particularly with therapies that increase CCS risk.
- As in the general population, the use of multimodality imaging should be guided by risk factor–weighted clinical likelihood model, other CV risk modulators, cancer prognosis, the specific anti-tumour treatment, and alternative therapies.
- Echocardiography is the first-line imaging modality to assess global and segmental ventricular function and to differentiate CCS from other CV conditions that may mimic it.
- For patients at low or moderate risk, anatomical imaging with CTCA is often preferred, allowing detection of both non-obstructive and obstructive CAD.
- In high-risk patients or those with established CAD, ischaemia testing is preferred, with exercise stress echocardiography, stress CMR, SPECT, or PET imaging according to local availability and expertise.

## Coronary microvascular dysfunction

Coronary microvascular dysfunction (CMD) has been described following antineoplastic treatment (i.e. alkylating agents, antimetabolites, taxanes, anti-VEGF, and antBCR-ABL TKIs) and RT. CMD is characterized by ischaemia and absence of obstructive epicardial CAD, and is associated with major adverse CV events, reduced quality of life,^68^ and worse outcomes in patients with breast cancer.^69^ The underlying mechanisms for chemotherapy-induced CMD include impaired vasodilation, prothrombic and pro-inflammatory effects, and microvascular spasm.^70–74^ RT induces CMD by endothelial damage to the microvessels due to pro-inflammatory, vasoconstrictive, and thrombotic effects.^75^

### Non-invasive cardiac imaging

CMD is characterized by impaired CFR and an abnormal index of microvascular resistance.^76^ When patients have clear symptoms of angina in the absence of obstructive CAD on CT or invasive angiography, the diagnostic suspicion of CMD usually arises. According to the 2024 ESC guidelines on CCS, in symptomatic patients with ANOCA/INOCA, medical therapy based on coronary functional test results should be considered to improve symptoms and quality of life.^39^ In addition, according to the ESC guidelines for CCS, in patients with refractory angina leading to poor quality of life and with documented or suspected ANOCA/INOCA, invasive coronary functional testing is recommended to define ANOCA/INOCA endotypes and appropriate treatment, considering patient choices and preferences.^39^ Although invasive testing is the reference method for CMD assessment in the general population, non-invasive method may be better suited for patients with cancer. Particularly in patients with a very poor overall prognosis and health, anti-anginal therapy could be started to obtain a diagnosis based on the benefit achieved following the therapy without resorting to further investigation. Anti-anginal drugs such as beta-blockers and calcium channel blockers could be the choice in this scenario; nitrate could also be added, even if they might have poor efficacy due to the occurrence of tolerance, and, if necessary, tricyclic medications and opioids.^39,76^

Among the non-invasive techniques, CFR may be assessed by stress Doppler echocardiography, which measures CFR from the maximal diastolic flow in the left anterior descending coronary artery (LAD) at rest and stress during adenosine/dipyridamole administration. This technique has been validated against intracoronary Doppler; however, it requires considerable expertise and adequate acoustic windows.^76^ Myocardial contrast echocardiography is also available for assessment, delivering MBF velocity and CFR.^77^

Further established modalities for non-invasive assessment of CMD are CMR and PET. Both modalities allow quantification of absolute MBF and the ratio of stress:rest MBF, providing the myocardial perfusion reserve, an index of CMD.

**Figure 9:**
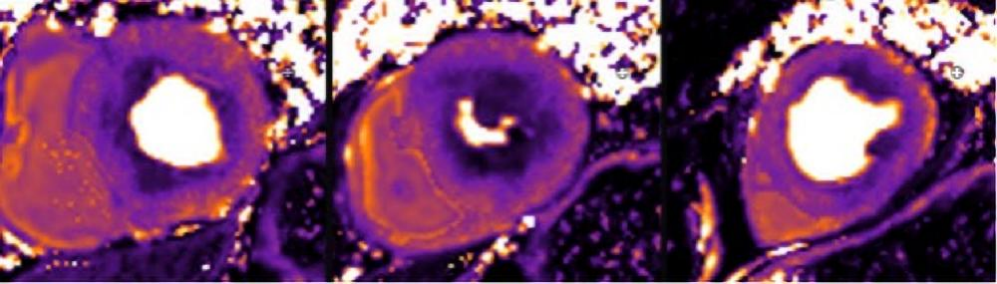
Peak stress perfusion cardiovascular magnetic resonance imaging maps of the base, mid and apex (from left to right), demonstrating subendocardial myocardial perfusion defect in a patient with coronary microvascular dysfunction (Courtesy of the School of Biomedical Engineering and Imaging Sciences at King's College London).

CMD can be assessed with CMR by dynamic contrast-enhanced first-pass myocardial perfusion following vasodilatory stress, such as with adenosine, and allows analysis of visual perfusion defects and fully quantitative MBF assessment^78^ The most common manifestation of CMD on CMR is a global subendocardial perfusion defect observed at peak stress (*Figure 9*). High-resolution CMR demonstrates good diagnostic performance for the assessment of CMD compared with invasive coronary flow measurements as the reference standard.^79^ Automated pixel-wise perfusion mapping technique can be used to detect not only significant CAD but also CMD, and to differentiate CMD from multi-vessel coronary disease.^64^

Similarly, PET can be used for the assessment of CMD through the administration of a perfusion radiotracer such as ^13^N-Ammonia, 82-Rubidium, or ^15^O-Water following vasodilator stress and rest.^80^ PET has also demonstrated good diagnostic value and prognostic value in patients with CMD.^81,82^

Key points

- CMD is common in patients with cancer, particularly in those who have undergone RT treatment.
- Although invasive testing is the reference method for CMD assessment in the general population, non-invasive method may be better suited for patients with cancer.
- Stress echocardiography with measurement of CFR in the LAD allows assessment of CMD. PET or stress CMR allow accurate quantitative assessment of the coronary microcirculation in all coronary territories with high diagnostic accuracy. The choice of non-invasive test should depend on local expertise and availability.

## Peripheral vascular disease

Several antineoplastic treatments, such as TKIs and radiation therapy, can cause peripheral vascular damage. Patients receiving RT of the neck have a markedly higher incidence of carotid artery stenosis compared to non-irradiated populations, with studies reporting stenosis in 21–86% of irradiated patients.^83^ RT significantly increases carotid intima-media thickness with increased stroke risk,^84^ although some studies suggest post-radiation thinning of the media due to medial fibrosis.^85^ High-dose radiation to the abdominal or pelvic regions may result in vascular stenosis in these regions, in particular renal artery stenosis.

Experimental models have demonstrated accelerated atherosclerosis in irradiated aortas, with severity increasing over time after irradiation.^86^ Radiation-induced aortic damage, which often manifests years after treatment, can lead to acute aortic complications.^87^ Though rare, aortic rupture is a recognized complication of radiation exposure, as well as direct aortic wall invasion by nearby tumours. Chronic aortic occlusion has also been reported following pelvic radiation therapy in childhood.^88^ Clinicians should be vigilant for these possibilities, arranging urgent care and assessment for patients with suspected acute and chronic aortic complications.

Chemotherapy, especially agents like anthracyclines and vascular endothelial growth factor inhibitors, can amplify these effects by inducing endothelial dysfunction, increasing oxidative stress, and promoting thrombosis.^89^

Patients receiving BCR-ABL tyrosine kinase inhibitors for chronic myeloid leukaemia, particularly nilotinib and ponatinib, have an increased risk of arterial vascular events such as PAD, stroke, and MI (2, 8–11% for nilotinib and 7–35% according to studies for ponatinib), risk varies according to dose intensity, older age, and CV risk.^12^

Moreover, many antineoplastic drugs can even induce Raynaud phenomenon and rarely acral vascular syndromes.^24^

### Non-invasive diagnosis

The mentioned complications underscore the need for baseline evaluation, monitoring during treatment, and long-term CV surveillance in cancer survivors, emphasizing the importance of incorporating CV risk assessment into cancer treatment planning.^5,90^

Patients with cancer, particularly those with head and neck cancer, fulfil several World Health Organization screening criteria, including a high burden of disease, the availability of acceptable non-invasive diagnostic tests, and the existence of effective preventive interventions. However, to date, no randomized controlled trials have evaluated screening for PAD in patients with cancer, and there is also no prospective evidence demonstrating that the detection of asymptomatic PAD in this population results in fewer CV events or improved survival.

According to the 2022 ESC guidelines on cardio-oncology, baseline imaging should be considered before radiation therapy, at 5 years following the therapy, and every 5–10 years thereafter.^5^ In patients with known arterial disease, follow-up may be offered at a shorter interval (e.g. yearly). Particularly in asymptomatic patients with a history of head and neck RT, carotid artery ultrasound scan should be repeated every 5 years starting at 5 years after radiation. Renal artery ultrasound should be considered in patients with a history of abdominal and pelvic radiation and worsening renal function and or hypertension.^5^

According to the 2024 ESC guidelines for the management of peripheral arterial and aortic diseases, ankle brachial index (ABI) is the recommended first-line measure to screen for the presence of PAD.^91^ In addition to ABI testing at rest, ABI can be measured after low-level treadmill stress, which is a simple procedure but increases the diagnostic yield. Routine clinical examination of arterial pulses in oncologic patients is also advocated. Duplex ultrasound is the first-line imaging modality. It is non-invasive, widely available, and provides information on location, plaque characteristics, and severity of vascular lesions through velocity criteria.^92^

MRI can provide a detailed assessment of the carotid arteries, aorta, and peripheral arterial system, the detection of luminal stenosis, as well as the detection of unstable or vulnerable plaque phenotypes in these regions.^93^

CT angiography (CTA) offers high-resolution evaluation of carotid artery stenosis and the presence of calcified plaques. However, it involves radiation exposure and the use of iodinated contrast agents; therefore, it is not used for screening. CTA and MR angiography have been found to have similar sensitivity; however, they differ in specificity, with CTA consistently showing higher specificity.^94^

CT imaging remains the gold standard for diagnosing acute aortic syndrome, with initial imaging ideally including non-contrast and post-contrast arterial phase CTA of the entire aorta, extending to the iliac and common femoral arteries. When structural issues are suspected, a delayed phase angiogram (60–90 s post-injection) can help detect slow contrast leaks. Delayed phase imaging may also assist in evaluating organ viability following an aortic syndrome. Long-term surveillance may be achieved with MRI or CT imaging techniques.

**Figure 10:**
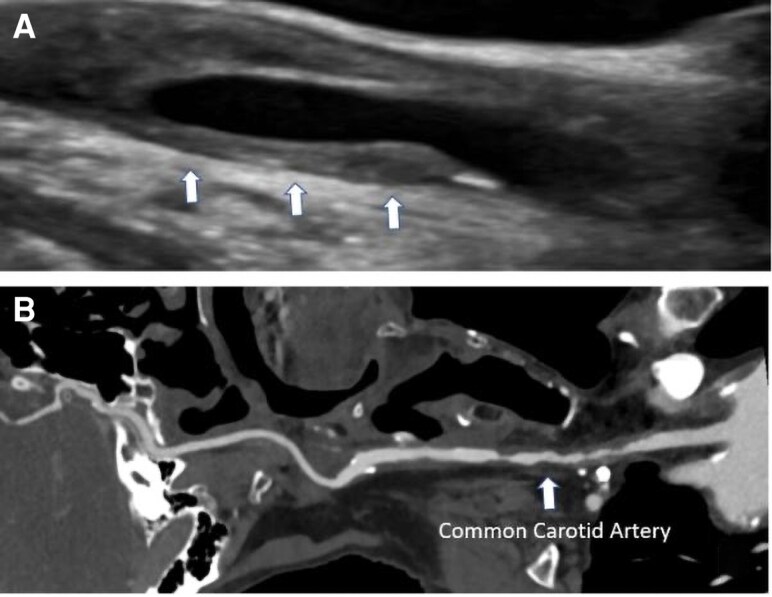
A 69-year-old male with a history of neck cancer, previously treated with surgical resection and neck radiotherapy, presented 13 years later for the consideration of coronary intervention. Ultrasound imaging (A) reveals diffuse non-calcified intimal-medial thickening (arrows) in the common carotid artery. Computed tomography angiography (B) shows diffuse non-calcified plaque disease in the common carotid artery, leading to multifocal severe stenosis.

*Figure 10* shows the case of carotid atherosclerosis in a patients underwent neck radiotherapy detected by ultrasound imaging (panel A) and confirmed by CTA (panel B).

**Figure 11:**
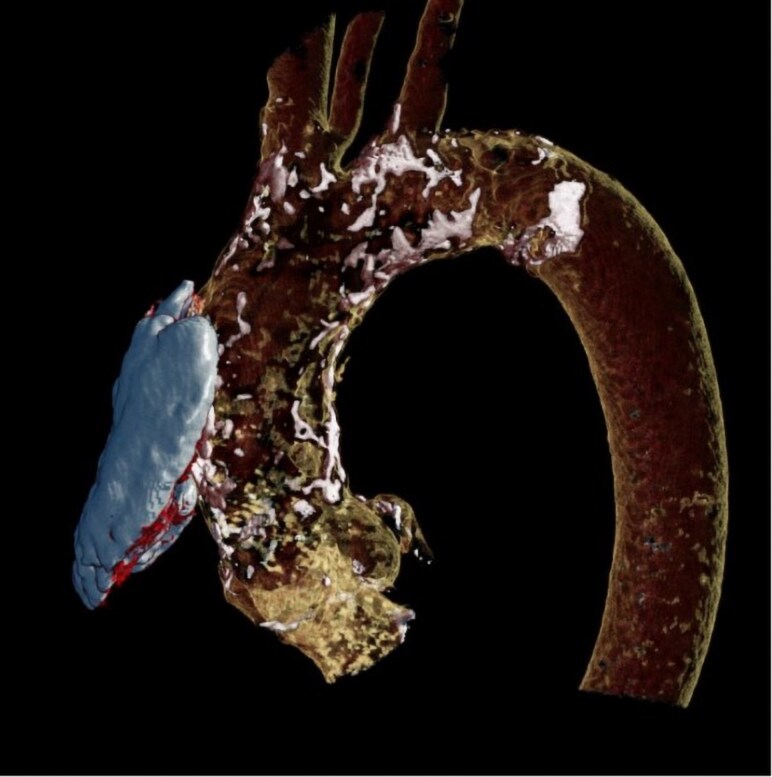
A 66-year-old female with a history of mantle radiotherapy for Hodgkin’s lymphoma underwent computed tomography angiography for planning a transcatheter aortic valve implantation as a result of radiation induced aortic valve stenosis. The imaging reveals heavily calcified anterior mediastinal lymph nodes (highlighted in blue) and diffuse calcification (highlighted in white) of the ascending aorta and aortic arch, indicative of radiation-induced aortitis.

*Figure 11* illustrates the radiation-induced aortitis revealed by CTA.

Key points

- Duplex ultrasound scan of carotid and peripheral arteries represents the first line imaging technique to address the presence of peripheral atherosclerosis and provides information for therapy optimization.
- CTA offers higher diagnostic accuracy but is not a screening tool given higher costs and exposure to radiation and iodinated contrast agent.
- CT is advised when acute vascular events are suspected.
- MRI has the advantage of high sensitivity without exposure to ionizing radiation, but lower specificity compared to CT.

## Conclusion

The role of multimodality imaging in detecting and managing coronary and peripheral arterial disease in patients with cancer receiving cardiotoxic antineoplastic treatments parallels that in the general population. However, the distinctive pathophysiological features and clinical presentations of this population require a high index of suspicion. Consequently, the use of cardiac imaging in these vulnerable patients is often customized to reflect clinical circumstances and individual patient characteristics.

## Data Availability

This study is based on previously published data. No new data were generated in support of this research. All data analysed during this study are included in the published articles cited in the reference list.
